# Genomics and disease resistance studies in livestock^☆^

**DOI:** 10.1016/j.livsci.2014.04.034

**Published:** 2014-08

**Authors:** Stephen C Bishop, John A Woolliams

**Affiliations:** The Roslin Institute & Royal (Dick) School of Veterinary Studies, University of Edinburgh, Midlothian EH25 9RG, UK

**Keywords:** Genetics, Epidemiology, Infection, Tolerance, Infectious pancreatic necrosis, Bovine tuberculosis

## Abstract

This paper considers the application of genetic and genomic techniques to disease resistance, the interpretation of data arising from such studies and the utilisation of the research outcomes to breed animals for enhanced resistance. Resistance and tolerance are defined and contrasted, factors affecting the analysis and interpretation of field data presented, and appropriate experimental designs discussed. These general principles are then applied to two detailed case studies, infectious pancreatic necrosis in Atlantic salmon and bovine tuberculosis in dairy cattle, and the lessons learnt are considered in detail. It is concluded that the rate limiting step in disease genetic studies will generally be provision of adequate phenotypic data, and its interpretation, rather than the genomic resources. Lastly, the importance of cross-disciplinary dialogue between the animal health and animal genetics communities is stressed.

## Highlights

• Factors influencing disease genetics studies are reviewed.
• Results are presented in detail for a viral disease and a bacterial disease.
• Breeding for resistance is often possible, but not always appropriate or necessary.
• Effective studies combine disease biology, epidemiology and genetics.
• Rate limiting step is likely to be provision of phenotypes rather than genotypes.

## 1 Introduction

Infectious disease is of major importance to livestock breeders for many reasons. For example, disease imposes a large cost on livestock production systems, with essentially all production systems being vulnerable to disease. Based on the direct costs of individuals diseases (e.g. Bennett et al., 2005), total disease costs have been estimated to be up to 20% of turnover in developed countries and as high as 35–50% of turnover within the livestock sector in the developing world. However, the true costs of disease are complex (Perry and Grace, 2009), depending on direct, indirect and intangible costs, which vary according to assumptions made about who is affected by the disease and the disease control measures. For example, infection may transmit across species. Several animal infections, such as bovine tuberculosis, pose zoonotic threats to human health, and diseases in one species may act as reservoirs for infections in other species. Additionally, there are pressures on breeders to address welfare issues and to reduce the reliance of production systems on control strategies such as extensive antibiotic and chemical usage, with regulation increasingly restricting antibiotic usage. For these reasons, rather than giving an actual cost, disease impacts are often considered to be a qualitative function of direct economic impact, industry and public concern, zoonotic potential and impacts on animal welfare and international trade (Perry et al., 2002, Davies et al., 2009).

Endemic infectious diseases pose particular challenges as these are diseases for which traditional disease control strategies, by their designation as endemic, are failing. Examples of worldwide importance include tick and nematode infestations, where there is widespread acaricide and anthelmintic resistance, respectively. Hence, alternative or complementary control strategies are required and breeding for increased host resistance to infection or disease is one such approach. Host genetic variation in disease resistance invariably exists, due in large part to the variability in host immune responses to infection (Bishop, 2010). Therefore, in principle, it may be possible to improve genetic resistance to most diseases, although ascertaining resistance phenotypes under field conditions can be challenging, as described below. For a subset of diseases, it may be both feasible to measure resistance traits on sufficient animals to determine genotypes for resistance and economically worthwhile to incorporate such traits into breeding goals. A detailed appraisal of infectious diseases that may be amenable to host genetic studies, and potentially selection for resistance, is presented by Davies et al. (2009). In cattle, for example, this study identified mastitis as a key disease, as had been expected, however it also identified tuberculosis and paratuberculosis as amenable diseases, and recent progress on both diseases has been substantial (see below for tuberculosis).

A rate-limiting step in breeding for disease resistance is the requirement to measure resistance phenotypes. This can be costly and logistically difficult, and it is a significant barrier to selecting for disease resistance. For this reason, disease resistance traits are an attractive target for genomic studies and are often the subjects of such studies. The benefit of the genomic approach is the ability to select animals using DNA-based selection without the need to expose them to infection in a challenge test, or for them to have been part of a natural epidemic. This can be achieved if major genes or QTL for resistance can be identified, or SNP-chip based genomic predictors (Meuwissen et al., 2001) of sufficient accuracy developed. Without DNA-based predictions, selection accuracy will depend on either routine challenge testing or continuous disease prevalence in the field, to enable calculation of EBVs based on expressed resistance phenotypes.

This paper aims to consider some of the issues associated with using genomics to understand disease resistance in livestock, and using genomic tools to assist in breeding for enhanced resistance. We consider basic concepts necessary to understand the issues encountered with this topic and, in additional to a broad-level literature review, we dissect two contrasting case studies, where resistance may be considered to be either ‘simple’ or ‘complex’.

## 2 Theoretical background

### 2.1 Resistance and tolerance

Terminology still causes confusion in this field. Firstly, the generic term ‘disease resistance’ is unfortunate as it implicitly confuses infection (invasion by a pathogen or parasite) with disease (the negative consequences of being infected). Resistance is best understood from an ecological consideration of the interaction between the host and the pathogen species (Grenfell and Dobson, 1995), may be defined as the ability of the host to exert some degree of control over the pathogen life cycle (Bishop and Stear, 2003, Bishop, 2012). This broad definition encompasses the many ways a host species may be more resistant (e.g., less likely to become infected, reduced pathogen proliferation once infected, reduced shedding or transmission of infection), and it also inherently recognises that resistance is usually relative rather than absolute. It also implies that altered resistance impacts on the population as a whole, as whilst some attributes benefit the individual host, other attributes (such as reduced transmission of infection) benefit other members of the host population.

**Fig. 1:**
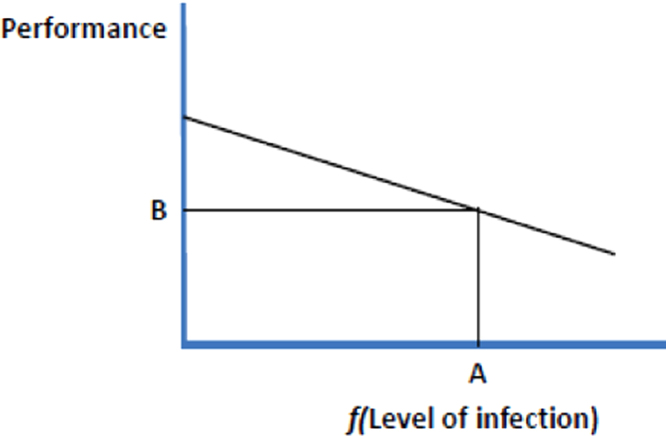
Definitions used in the paper are: *Resistance* is the ability of the host animal to exert control over the parasite or pathogen lifecycle; *Tolerance* is the net impact on performance of a given level of infection; *Resilience* is the productivity of an animal in the face of infection. The figure (from Bishop, 2012) shows a schematic representation of performance and level of infection (or some function that linearises the relationship between level of infection and performance). The regression slope represents *Tolerance*, point A indicates *Resistance* and point B represents *Resilience*.

Tolerance is different from resistance, and is discussed in depth by Doeschl-Wilson et al. (2012), and other papers in the Special Topic in Frontiers in Livestock Genomics (2012) on tolerance. Again using the definitions specified by Bishop (2012), tolerance may be defined as the net impact on performance of a given level of infection, i.e. the regression of performance on (a function of) pathogen load. A related concept, resilience, may be defined as the productivity of an animal in the face of infection. Whereas resistance implies a host exerting a deleterious influence on the fitness of the pathogen, hosts with a greater tolerance are those able to maintain a greater fitness as pathogen load increases. Definitions are presented diagrammatically in Fig. 1.

As a trait defined at the individual animal level, tolerance presents a number of difficulties and it also has a number of inherent assumptions that often seem to be ignored. Firstly, given that it describes the change in performance as pathogen load changes, individual animal performance has to be measured at different levels of pathogen burden, whilst at the same time keeping all other husbandry and environmental conditions as constant as possible. For most diseases this is problematic, especially as immune responses alter with continuing exposure to infection. In reality, it can probably only be measured at the individual animal level for traits expressed repeatedly through life and for diseases where the immune memory is weak. Some infections in lactating animals may fall into this category, for example mastitis in dairy ruminants or nematode infections during the periparturient period of compromised immunocompetence.

The issue of requiring different infection levels can be overcome to some extent by considering host genetics at the family level, so a sire׳s genetic merit can be observed as a reaction norm, with offspring with different pathogen burdens providing the necessary repeated observations. But even in this case, family size has to be sufficient to overcome issues of between-sib variability. However, a bigger issue with tolerance as a trait and a breeding goal is that it is only expressed by infected animals and, to be useful, requires the disease to be at high prevalence (Bishop, 2012). Consider the case with a moderate prevalence and susceptibility to infection to be a genetically controlled trait. The animals that are not infected are the most resistant to infection (in terms of susceptibility to infection per se) and therefore the most interesting to the breeder; however they will not have a phenotype useful for assessing tolerance. It is only the least resistant animals that provide information on tolerance. Under this scenario, unless prevalence is high, i.e. approaching 1.0, tolerance describes attributes of the wrong animals for the breeder, as by definition it focusses on the least resistant rather than the most resistant animals.

Some of the measurement issues associated with tolerance may be overcome by the novel interpretations of tolerance suggested by Doeschl-Wilson et al. (2012), as the outcome of host-pathogen interaction trajectories. These are trajectories which describe the joint changes in animal infection level and performance over time, which account for time-dependent impacts of infection and lend themselves to mathematical analyses. However, without the use of sophisticated approaches such as this, and recording sufficient to implement it, it is our belief that in most cases, particularly for diseases where prevalence is substantially less than one, genomic studies should focus on resistance traits rather than tolerance. In cases where all animals are infected, then resilience (performance in an infected environment) becomes a useful concept.

### 2.2 Interpreting and analysing field data

In order to obtain sufficient data to quantify genetic variation in resistance and to perform genomic studies it is often necessary to use field data. Whilst such data can be extremely informative, and natural disease outbreaks can provide data cost-effectively, there are a number of sources of environmental noise that potentially mask the genetic signal. These include incomplete exposure to infection, imperfect diagnostic tests and variable infection pressures over time and between environments. These influences will all tend to reduce heritabilities and the power to detect SNP associations, as outlined by Bishop and Woolliams (2010a) and Bishop et al. (2012). A broad summary of the main issues are given here.

Incomplete exposure to infection results in some animals not having the opportunity to express their resistance genotype. Therefore, uninfected animals will comprise individuals that may truly be resistant (at the level of challenge they have encountered) or animals that have yet to be exposed to an infectious dose of pathogen. Assuming that it is not possible to distinguish between these two categories of animals, incomplete exposure biases both estimated SNP effects and heritabilities downwards, with the former reduced by a factor ε, where ε is the proportion of the population exposed to the infection, assuming that exposure is an all or none event (see below). Furthermore *ε* will change continuously during an epidemic, and accounting for epidemic dynamics whilst estimating quantitative genetic parameters remains computationally challenging (Lipschutz-Powell et al., 2014).

Collection of field data requires diagnosis of the infection (or disease) state of an animal, with all diagnostic tests being described by the concepts of specificity and sensitivity. Specificity (*S*_*p*_) is the probability that a truly *healthy* individual is classified by the diagnostic test as *healthy* and sensitivity (*S*_*e*_) is the probability that a truly *diseased* individual is classified by the diagnostic test as *diseased*. This parameterisation of the 2×2 classification of true status and diagnosed status is universal in epidemiological theory, rather than the alternative classification of true and false test outcomes. If either *S*_*p*_ or *S*_*e*_ is less than one, then observed prevalence (*p*′) will differ from true prevalence according to the following regression: $p\prime =(1-S_{p})+(S_{p}+S_{e}-1)p$. As with incomplete exposure, imperfect diagnosis will reduce both heritabilities and estimated SNP effects, with the SNP effect biased downwards by the factor $\left(S_{p}+S_{e}-1\right)$ (Bishop and Woolliams, 2010b, Bishop, 2012).

The consequences of incomplete exposure to infection and imperfect diagnosis are simply that genetic signals get diluted and the power to quantify genetic effects is reduced. These factors probably lie behind the commonly-held belief that disease resistance traits are lowly heritable, an observation that flies in the face of the near-ubiquitous variation seen in immune-related genes and in immune responses (Bishop, 2010). Therefore, the identification of a genetic signal for resistance under field conditions most likely indicates an underlying genetic control that is much stronger. Consequently, the opportunities for studying genetic resistance to disease, and even selecting for increased resistance, may be somewhat greater than is apparent from low observed heritabilities.

The considerations so far have considered only ‘static’ data, i.e. they have ignored the time-dependent changes in infection pressure. The impacts of variable infection pressures on genetic parameter estimation are complex and have yet to be fully elucidated. They will vary according to whether infection pressure is presumed to be ‘constant’ but different in different circumstances/environments, or whether it varies dynamically during an epidemic. An example of the former is discussed below, in the salmon disease case study. The latter case is addressed by Lipschutz-Powell et al. (2014), with analytical properties of the estimates of genetic effects presented by Pooley et al. (2014).

### 2.3 Appropriate experimental designs

The ideal experimental design for detecting genetic variation in resistance and for identifying SNP associations or developing genomic predictors of resistance would exploit continuously varying phenotypes measured on animals subjected to identical environmental and challenge conditions. Such circumstances, where challenge conditions are deliberate and standardised, are rare and are most likely to be feasible for studies in fish, where several examples do exist (Wetten et al., 2007, Moen et al., 2009, Houston et al., 2010, Gheyas et al., 2010) or chickens (e.g., Pinard-van der Laan et al., 2009). Under some circumstances it has been possible to achieve this situation for terrestrial mammals, e.g. the PRRSV challenge experiments described by Boddicker et al. (2012), however these studies generally require large-scale funding coordinated between several partners and hence are relatively uncommon.

However, even in the context of challenge tests there is now a recognition that typical studies address genetic variation in susceptibility to infection but do not address the possible existence of genetic variation in infectiousness (Lipschutz-Powell et al., 2012). Infectiousness has an indirect genetic effect on the population and, unlike competition effects among animals (Bijma, 2010), it is dynamic. Its detection depends primarily upon detecting variance in the speed of epidemic development among groups. This is both demanding of data, requiring “replicated” epidemics and computationally demanding. The novelty of this area means that optimum challenge designs for detecting such variation have yet to be defined. However, initial results are given by Pooley et al. (2014) who present analytical solutions for prediction accuracy in the case of major gene effects and propose novel Bayesian inference approaches for estimating such effects. In summary, unless infectiousness can be measured directly, it is likely to pose estimation problems.

Disease resistance studies more commonly use opportunistic ‘harvesting’ of data either from epidemics, such as bovine tuberculosis (bTB) outbreaks (e.g., Bermingham et al., 2013) or from endemic diseases such as mastitis or nematode infections. Even in this situation distinct differences are seen between endemic and epidemic diseases. For the two endemic diseases mentioned, phenotypes can be captured by measurements which show continuous variation, whereas for the epidemic diseases the phenotype is more often a binary variable, i.e. infected/diseased or not.

One of the issues faced when using data from an epidemic, particularly when the outcome is a binary variable (affected or not), is the choice of animals to include in the dataset, and hence to genotype. Ideally, one would sample all animals from a cohort, or take a random sample with affected and unaffected animals sampled in proportion to the disease prevalence. However, there are several factors to consider when making such decisions. Firstly, if prevalence is low, then sampling many unaffected or control animals can be perceived as wasteful of resources when compared to standard case-control designs which maximise the power of a contrast. However, case-control designs make estimation of, or correction for, non-genetic factors difficult as the sampling has been non-random and, hence, the effects of both genetic and non-genetic factors will be incorrectly estimated. Secondly, definition of control animals may be problematic, especially if exposure to infection is unknown or if diagnostic test sensitivity is low. In either case animals will be misclassified and, combining the two concepts, the downward bias in estimated SNP effects will be $\varepsilon (S_{p}+S_{e}-1)$.

The problem of control definition has often been avoided in human genetics studies using the so-called Wellcome Trust design (e.g. Browning and Browning, 2008), in which cases are compared against a reference population average sample. In cases where disease prevalence is low, or diagnostic test sensitivity (or ascertainment of cases) poor or exposure probabilities low, then true controls are unlikely to differ greatly from a random sample from the population, and the two experimental designs converge. The Wellcome Trust design may also be advantageous in situations where large numbers of ‘population average’ animals (for the trait of interest) have already been genotyped, an obvious example being the large numbers of Holstein dairy animals genotyped as part of genomic selection programs. But it is appreciated that apart from the case of Holstein cattle, the sub-population structure often seen in livestock will make it difficult to define appropriate ‘population average’ animals. However, in cases where true controls (i.e., uninfected animals that have been exposed to an infectious dose of pathogen) can be defined with some accuracy, then alternative experimental designs have been proposed which may have greater power (Bishop et al., 2012). Quite simply, animals could be sampled to maximise their expected genetic differences in resistance to the disease. Therefore, cases could be preferentially sampled from cohorts with a low force of infection (therefore more susceptible) and controls preferentially sampled from cohorts with a high force of infection (therefore more resistant). However, the properties of this design are unknown, and potentially it creates risks in terms of unobserved risk factors and hidden genetic structure, and research is required to quantify the balance between extra power and greater risk of unknown factors biasing the results.

## 3 Overview of disease genomic studies

The purpose of this paper is not to tediously review all genomic studies looking at disease resistance in livestock. Rather, we wish to draw attention to a few salient points. Firstly, genetic variation in disease resistance has been observed for many diseases (Bishop, 2010), and most likely variation would be seen for all diseases, given appropriate experimental designs. Published examples exist for every class of infectious agent, ranging from TSE agents, through viruses, bacteria, protozoa to worms, flies and ticks, and also for all major livestock species, including several aquacultural species.

Predictably, genome scans by LD are now increasingly popular, although these studies tend to require a larger sample sizes than conventional heritability or within-family QTL studies. A major focus has been on major endemic diseases, notably bovine tuberculosis (see below) and paratuberculosis (e.g., Kirkpatrick et al., 2011, Minozzi et al., 2012), PRRS in pigs (Boddicker et al., 2012), and nematode infections in sheep (Kemper et al., 2011, Sallé et al., 2012, Riggio et al., 2013). These are diseases that the respective livestock industries tend to live with, accepting the ongoing costs, and for which control measures, including vaccination, have been unable to eliminate the disease. The disease that is notably missing from this list is bovine mastitis. In this case, industry-wide recording of somatic cell count and clinical mastitis, combined with widespread SNP-chip genotyping of dairy bulls, has led to the rapid implementation of genomic selection over the last six years with relatively few genome-wide association studies (albeit with notable exceptions, e.g. Sahana et al., 2013).

We now illustrate issues and success with genomic disease resistance studies by considering two case studies that we have been involved with. These two examples highlight most of the issue discussed above.

### 3.1 Case study for simple inheritance: infectious pancreatic necrosis

#### 3.1.1 Background

The salmon viral disease infectious pancreatic necrosis (IPN) is one of the best case studies of the application of genomic technologies to an infectious livestock disease and one of the first implementations of DNA-based selected. Parallel and simultaneous research programs in Norway and Scotland came up with almost the same findings, as described below, and each served as an independent validation of the other study. Outcomes from the research are now routinely incorporated into the breeding programs of the two associated breeding companies, AquaGen in Norway and Landcatch Natural Selection Ltd in Scotland, with the result that IPN is no longer a disease of major concern for these two companies.

The IPN virus is a double stranded RNA virus (i.e., a birnavirus) which has been endemic in the European salmon aquaculture industry, affecting both juvenile fry and seawater stages of the salmon lifecycle. In freshwater, juveniles are most susceptible before the immune system is fully developed, and hatchery mortality losses in fry around first-feeding can reach 70% or more (Roberts and Pearson, 2005). In seawater, a clearly defined window of susceptibility coincides with the stress of smolting and seawater transfer, at approximately 15 months post-hatching, with mortality ranging widely, i.e. anywhere from zero to >90% (Moen, 2010).

#### 3.1.2 Establishing genomic control of resistance

Salmon reproductive biology allows creation of many large full-sib families, which can then be monitored in either field studies or deliberate challenge studies. This allows for robust genetic and genomic studies, and it allows secondary questions to be addressed as described below. These large-scale studies established heritable variation in IPN survival at the smolt stage under field conditions (Guy et al., 2006, Guy et al., 2009; mean *h*^2^=0.43), and at the fry stage under challenge conditions (Wetten et al., 2007; mean *h*^2^=0.31). Microsatellite-based QTL studies performed on salmon smolts under either natural challenge (Houston et al., 2008) or deliberate challenge conditions (Moen et al., 2009) demonstrated that nearly all the observable genetic variation (in IPN survival) could be attributable to a single QTL on linkage group 21. Follow-up studies on fry, using deliberate challenge techniques on large numbers of fish from a wider range of families, confirmed that the same QTL also largely controlled IPN survival in the fry (Moen et al., 2009, Houston et al., 2010, Gheyas et al., 2010). Further, these later studies reduced the confidence interval for the mapped QTL from ca. 10 cM to much smaller intervals, ca. 3 cM.

The cross validation of results and the finding of similar effects in two disparate lifecycle stages was critically important for promoting confidence in the results, increasing precision, and also for allowing both windows of susceptibility to be addressed simultaneously. Host-pathogen interactions may be postulated to be quite different in lifecycle stages where the immune responses to infection differ. Furthermore, the fact that the same QTL affected both lifecycle stages enabled subsequent experimentation and phenotype scoring to be performed in the logistically simpler fresh-water fry stage, rather than in sea-water cages. Both the Scottish and Norwegian studies showed a frequency of the putative resistance allele to be ca. 0.25 in unselected fish (Houston pers. comm., Moen, 2010). The results from the studies allowed marker-assisted selection (MAS) to be implemented by the breeding companies involved with the research, with a single round of selection in the Norwegian population increasing the frequency from 0.27 to 0.44 (Moen, 2010). Initially this was linkage-based MAS with the requirement for reassessment of linkage phase between markers and the putative underlying polymorphism every generation. A more effective and sustainable MAS strategy would ideally utilise population-wide LD between marker and causative mutation.

#### 3.1.3 Fine mapping resistance markers

As pointed out by Moen et al. (2009), implementation of MAS based on LD would require a greater density of markers than was available at the time of the studies. The absence (in 2010) of a reference genome or a dense SNP chip made progress difficult. This problem was initially solved through the use of RAD sequencing (Baird et al., 2008) applied to families previously used for confirming the IPN resistance QTL in fry (Houston et al., 2010). Using SNP discovery by sequencing in families and individuals with well-defined QTL genotypes, it was possible to identify SNP markers in complete linkage with the putative QTL genotype, in the previously-genotyped families (Houston et al., 2012). After filtering, 11 SNPs were genotyped across 10 families used in the QTL study, and the two most significant SNPs were then genotyped on ca. 4000 fish from 200 related families from the same (discovery) cohort, and ca. 5000 fish from 200 families from a different (distantly related, i.e. validation) cohort. Results were consistent across both cohorts, with mortalities close to 10% for homozygous resistant fish, 20–25% for heterozygous and >50% for homozygous susceptible fish (Houston et al., 2012).

The outcome of the research undertaken by both groups of researchers is that markers now exist that are in close population-wide LD with the causative mutation. Hence, these markers can be used directly and reliably in breeding programs. It is important to note that whilst the QTL studies of Houston et al. (2010) appeared to show zero mortality in homozygous resistant fish, some mortality is observed in fish of this genotype in the wider population. One interpretation of this result is that the markers are not in complete LD with the causative mutation, although other interpretations are explored below. At the time of writing, neither the actual causative mutation nor the mechanism of resistance is known, despite considerable research effort. However, selection for resistance has been successful even without knowledge of the causative mutation.

#### 3.1.4 Inferences on the nature of resistance

Whilst the genetic control of IPN resistance is strong and consistent, there are still unexplained nuances which may be explicable when the disease epidemiology is considered. For example, estimated heritabilities for IPN-related survival of salmon in seven independent seawater localities containing the IPN virus (Guy et al., 2009) showed a strong pattern of increasing as prevalence of mortality increased, even after correcting for the binary nature of the data by transforming to the underlying liability scale. However, when the assumption was made that prevalence is a proxy for relative exposure probability (i.e., *ε*), and the heritabilities were corrected for exposure as described by Bishop and Woolliams (2010a), then the relationship between prevalence and heritability of resistance disappeared. Notably, the heritability of exposure-corrected heritability resistance coalesced around a value in excess of 0.9, which is consistent with the QTL controlling most of the variation in IPN-dependent mortality. Hence, the assumption of prevalence being a proxy for relative exposure probability appears valid.

Secondly, comparing the different experiments within which QTL have been (fine) mapped (i.e., Moen et al., 2009, Houston et al., 2010, Gheyas et al., 2010) it may be observed that in some populations the resistance locus is additive; whereas in others it is apparently dominant. Further, this apparent mode of inheritance seems to vary with prevalence of mortality, with lower mortality trials leading to apparent dominance and trials with intermediate mortality leading to additive effects. A rationale for this outcome was provided by Bishop and Woolliams (2010b), in which the consequences of dose-dependent expression of resistance were explored. This is intrinsically related to the concept of exposure, in which resistance may be re-defined as the dosage level at which an individual becomes infected. When dose–response curves tend asymptotically towards zero mortality for negligible infectious doses and towards a high mortality for overwhelmingly high infectious doses, the apparent mode of action of resistance is a function of the infectious challenge level. Under field conditions, the infectious dose corresponds to the extent of the epidemic, sometimes termed the force of infection. Thus, ignoring possible impacts of other QTL with smaller effects, one may expect to observe dominant, additive or recessive effects depending upon the disease epidemiology even when the underlying liability is completely additive.

### 3.2 Case study for complex inheritance: bovine tuberculosis

#### 3.2.1 Background

Whilst IPN provides an elegant case study for understanding the inheritance of resistance largely controlled by a single locus, the full complexities of understanding and dissecting resistance, when resistance is seemingly complex, are well illustrated for bovine tuberculosis (bTB). bTB is a bacterial disease caused by the bacterium *Mycobacterium bovis*, and it is an endemic disease with zoonotic potential in many parts of the word, notably in the United Kingdom (UK) and the Republic of Ireland (RI). Here, the zoonotic threat can be controlled by pasteurisation, but despite five decades of efforts to control bTB, the disease remains an ongoing challenge. The primary means of control is by means of compulsory testing of cattle followed by slaughter of test-positive animals, with total costs exceeding £275 million in 2010/11, alone (Abernethy et al., 2013). The impact of the disease is much larger globally, and Perry et al. (2002) ranked bTB as the fourth most important disease in developing countries, where *M. bovi*s causes an estimated 10–15% of human TB cases (Michel et al., 2010). Because of its importance, its endemic nature and the continued failure of mainstream control strategies, bTB emerges as a strong target disease for genomic studies of host resistance.

Research into bTB resistance in the UK and RI has benefitted from parallel research programs in the same way as seen for research into IPN resistance in salmon, with expertise and concepts shared, with the two sets of results essentially serving as cross validations and, potentially, with data sharing.

#### 3.2.2 Capturing data from the field

Unlike salmon, it is not possible to do large-scale challenge studies for bTB resistance. Therefore it is necessary to capture data from the field, specifically to harvest (with permission) data arising from statutory surveillance activities. Separately in England and Wales, in Northern Ireland (NI) and in RI all cattle herds are tested regularly (e.g., annually) for the presence of bTB in the herd. Typically, cattle are tested using a skin test, the single intradermal comparative cervical tuberculin (SICCT) test, with positive animals slaughtered and examined for clinical evidence of disease or infection. The presence of skin test positive animals then triggers more intensive testing which continues until the herd is deemed bTB free. However, data arising from this approach has inherent challenges, because both the skin-test and the abattoir inspection diagnoses are imperfect, and variable exposure to infection creates difficulties for phenotype definition and genetic studies.

Consider the diagnostic properties of the skin test and abattoir diagnoses of visible lesions. Both are effective at diagnosing affected herds, but they are poor at diagnosing the state of individual animals. Inevitably there has to be a trade-off between *Se* (a function of false negatives) and *Sp* (a function of false positives), and the test cut-off levels can be calibrated to alter this balance. Under field conditions, whilst the skin test has a high *Sp* (i.e., >99%), its *Se* is somewhat lower, being in the vicinity of 0.7 (De la Rua-Domenech et al., 2006), and possibly as low as 0.55 (Neill et al., 1994, Bermingham et al., 2011). In other words, close to 50% of truly infected animals may be missed with a single skin test per animal. Efficiency of diagnosis in the abattoir is of equal concern; whilst there are many reasons why infected animals may be missed when scanning for visible signs of infection, *Se* may be below 30% (Bermingham et al., 2011). Therefore, although one may have confidence that animals diagnosed as infected most probably are infected, many true cases will be misclassified unless multiple observations are available, and this incorrect classification will bias both heritability and SNP effect estimates, as described above in Section 2.3.

Exposure to infection is equally problematic, however steps can be taken in both trait definition and experimental design to address this challenge. Firstly, whilst bTB is an endemic disease in the UK and RI, not all herds are affected at any point in time. Therefore, data should be restricted to herd cohorts within which cases occur and, ideally, there should be two or more cases to increase the probability that it is a true bTB outbreak being observed. Single cases within a herd risk being either a rare false positive or an animal imported into the herd whilst infected. Secondly, the problem of time-dependent exposure can be reduced by classifying a case as an animal that is *ever* diagnosed as positive, whereas controls (unaffected) are those from outbreak herds that are *never* diagnosed as positive. The principles of this ‘ultimate fate’ model were employed by Bermingham et al. (2009) and Brotherstone et al. (2010) in their successful studies of the heritability of bTB resistance. This approach may lose information on susceptibility that may be related to the order in which animals become infected, but it reduces the impact of the imperfect testing.

#### 3.2.3 Genetic and genomic control of resistance

Heritable variation for bTB resistance within the RI and UK Holstein dairy herds has been established by Bermingham et al. (2009) and Brotherstone et al. (2010), respectively. Heritabilities for ultimate fate, based on skin test results, were 0.14 and 0.15, and for presence/absence of visible abattoir lesions heritabilities were 0.18, in both datasets. When corrected for imperfect diagnostic sensitivities, these values rose to ca. 0.20–0.25. Similarly, a case-control study from NI, in which cases were both skin test and abattoir lesion positive and controls were ‘never-positive’ cows from affected herds, yielded a heritability of 0.21 (Bermingham et al., 2014).

Genome wide association studies have now been completed on the RI and NI datasets described above (Finlay et al., 2012, Bermingham et al., 2014), using the 50k and high density SNP chips, respectively. In both cases some evidence of loci affecting liability to infection was reported, however the strong impression gained from these data was that resistance was a polygenic phenomenon, controlled by many loci. Hence, the genetic control of bTB resistance may be considered to be truly complex.

In circumstances where genetic control of a trait is complex, genomic selection may be preferred to conventional MAS based on individual loci. Following this reasoning, Tsairidou et al. (2014) demonstrated using the case-control data from NI that genomic prediction of bTB resistance is possible in principle, with prediction accuracies closely reflecting expected values (Daetwyler et al., 2008) given the dataset size, numbers of markers genotyped and presumed effective population size of the Holstein breed. Indeed in situations such as bTB, where the disease is endemic but not present in all herds/flocks, genomic selection is advantageous. Whilst conventional pedigree-based EBV estimation is possible, it relies on continual data collection from affected cohorts of animals, which is logistically difficult, and EBV accuracies for animals only distantly related to those in affected cohorts will be poor. Once calibrated with sufficient data from the reference population, genomic selection overcomes many of these problems, as it allows EBV estimation for animals distantly related to those with phenotypes and it facilitates data capture from herds without pedigree recording. However, in the short term, genomic selection is likely to be only feasible within the Holstein breed, as it will take more time to create suitable reference populations for other breeds.

#### 3.2.4 Lessons learnt and next steps

bTB has served as an interesting case study for situations where the data is noisy and where genetic control of trait variability is complex. Much of the thinking behind the papers of (Bishop and Woolliams, 2010a, Bishop and Woolliams, 2010b) came in response to challenges interpreting bTB data and the need to frame quantitative genetic concepts in a language more familiar to disease control experts. Even given many vagaries in the data, coherent genetic messages can still be obtained and routes to implementation (i.e., breeding for increased resistance) can be devised.

Challenges remain in the interpretation and analyses of bTB data. Firstly, whilst a framework for interpreting bTB resistance traits has been laid out, the true impact of subtle differences in trait definition on genomic predictions have yet to be fully explored. Secondly, many concerns exist within the animal health community related to selection of animals on the basis of a diagnostic test which is a response to infection; these concerns have previously been encountered with selection for mastitis resistance based on somatic cell count. The issue is that selection on a response to infection may potentially alter the actual response to infection as opposed to resistance to infection, and hence simply alter the properties of the test without necessarily changing resistance. This concern can be addressed through analyses of actual test values in existing datasets, interpretation of the magnitude of heritabilities for different trait definitions, and prediction of likely selection intensities and responses to selection. These issues are a focus of current research using bTB test data.

Lastly, and importantly, the impact of genomic selection for bTB resistance on prevalence of disease has yet to be ascertained. This will depend largely on the basic reproductive value (*R*_0_) for the disease; if *R*_0_ values are close to the threshold value of 1.0, then small changes in resistance could have large impacts on realised disease prevalence, whereas for higher *R*_0_ values selection may have little effect on prevalence. This issue is made more complex in the case of bTB due to the presence of wildlife hosts that serve as a reservoir transmitting infection, notably the badger in the UK and RI. Addressing this issue will require disease modelling combining host genetics and epidemiology, possibly including transmission of infection to and from the reservoir hosts. In summary, it can be seen that the rate limiting steps for pushing forward our understanding of the genomic control of bTB resistance would appear to lie more with phenotype availability and interpretation, than with availability of suitable genomic data. In fact, this final conclusion can probably be applied to most infectious diseases of interest to animal geneticists.

## 4 Conclusions

Genetic and genomic studies of disease resistance are becoming ever more widespread, with many published examples of genetic variation in resistance. This has translated into some notable examples of success in terms of describing and understanding the genetic control of between-animal differences in resistance, and also in the utilisation of these results to breed animals for increased resistance. However, use of genetic or genomic information must be considered in the broader context of both the breeding goal and the conventional strategies used to control the disease. If the disease is of little importance to the breeder or producer, or if it is satisfactorily controlled by other means, then there may be little point in attempting to breed for increased resistance. Consequently, most effort should be placed on costly endemic diseases, for which control by other strategies is proving difficult. The two example diseases considered here, IPN and bTB, fall into this category. Both represent cases where considerable progress has been made, but challenges still remain in terms of full understanding of the obtained results and optimal utilisation strategies.

Feasible data collection methods will depend largely on the reproductive capacity and net value of the individual host animal. Where the reproductive capacity is high (e.g., fish or chickens), it may be possible to perform deliberate challenge experiments on sufficient animals for genomic studies, but for the most part it will be necessary to capture field data from naturally infected populations. In this case, factors such as challenge levels, exposure probabilities and diagnostic test sensitivities and specificities will influence the estimated heritabilities, genetic marker effects and genomic predictions. The influences of these factors are discussed above and, in general, they will tend to add noise to the data and hence mask true genetic signals. There remain issues to be fully understood regarding optimal experimental designs and optimal data analysis procedures. Mostly, the knowledge gaps relate to handling and interpretation of phenotypic rather than genetic information. This conclusion stresses the importance of health data collection, as well as access to and sharing of existing data. Such data are a pre-requisite for powerful genetic studies.

Efficient implementation routes, i.e. effective breeding for enhanced resistance, will depend on the disease epidemiology. For example, if the disease is widespread with a high prevalence then selection based on phenotypic information is feasible, as has been seen for nematode infections and mastitis in ruminants. However, if disease outbreaks are sporadic or if prevalence is low, then DNA-based selection will be preferable. For example, for the case of bTB discussed above, data collected from national surveillance programs can be used to estimate breeding values for resistance; however the reliability of these EBVs will be poor for animals distantly related to those in an outbreak. In such cases, genomic predictions of resistance will greatly assist the implementation of breeding for resistance.

Lastly, breeding for disease resistance is a multi-disciplinary activity. Our own experiences strongly reinforce the need for widespread dialogue between geneticists and animal health experts, and the need to incorporate concepts from disease biology and epidemiology into animal genetics, and vice versa. Both communities must understand the goals and techniques used by the other community.

## Conflict of interest

There are no conflicts of interest (financial, personal or other relationships with people/ organisations) that could inappropriately have influenced our work.
